# Art therapy and neuroscience: evidence, limits, and myths

**DOI:** 10.3389/fpsyg.2024.1484481

**Published:** 2024-10-02

**Authors:** Christianne E. Strang

**Affiliations:** Department of Psychology, University of Alabama at Birmingham, Birmingham, AL, United States

**Keywords:** mirror neurons, neuroplasticity, hemispheric asymmetry, art therapy research, interoception

## Abstract

The evidence base for the effectiveness of art therapy continues to grow, even as a mechanistic understanding of how art therapy works remains limited. One promising avenue for increasing our understanding of how and why art therapy works is through the lens of neuroscience. A neuroscience-based approach to art therapy provides opportunities for improving understanding of the neural processes that underlie the complex interaction between perception, cognition, emotion and behavior that play out in the art therapy process. Understanding how therapeutic change occurs can result in improved treatment and better outcomes for clients. However, it can be tricky to connect art therapy and psychological theory directly to neural responses. The purposes of this perspective are to provide an overview of the current evidence and limits of neurobiological concepts of neuroplasticity, mirror systems, and interoception as applied to art therapy practice, and to provide updated information about outdated concepts that are still actively used in clinical practice. Critical analysis and understanding of the current scientific knowledge base can then be used to guide art therapy practice and support the development of hypothesis-based research to determine the primary mechanisms that drive the observed effects of art therapy interventions.

## 1 Introduction

Humans have engaged in art making for thousands of years, and one can make the argument that creativity is a hallmark of being human (Morriss-Kay, 2010; Zaidel, 2013). Humans use art and creativity for communication, self-expression, self-healing, and creating community. Therapeutic art is the structured use of the human creative impulse to enhance emotional well-being, while the profession of art therapy has its roots in the US and Britain in the 1940s (Vick, 2003; Waller, 2013). Art therapy as practiced by trained and credentialed art therapists “enriches the lives of individuals, families, and communities through active art-making, creative process, applied psychological theory, and human experience within a psychotherapeutic relationship” (American Art Therapy Association, n.d.).

There are roughly 80 years’ worth of anecdotal evidence and case studies showing that art therapy is helpful on an individual basis. There are also a growing number of qualitative and quantitative studies on the effectiveness of art therapy across populations and treatment settings (Dunphy et al., 2018; Jiang et al., 2020; Kaimal et al., 2018; Masika et al., 2020; Newland and Bettencourt, 2020; Walker et al., 2018; Xu et al., 2020). Thus, the evidence base for the effectiveness of art therapy continues to grow, even as we do not really know how or why it is effective.

One promising avenue for increasing our understanding of how and why art therapy works is through the lens of neuroscience (Khalil et al., 2019; Khalil and Demarin, 2023; King et al., 2019). This approach is especially promising because art-making directly engages visual and tactile sensory inputs that are integrated with emotion, memory, and cognition, that in turn affect voluntary and involuntary motor outputs (Kaimal et al., 2018; Kandel et al., 2021; Magsamen and Ross, 2023; Pénzes et al., 2023). The framework of the Expressive Therapies Continuum (ETC) explicitly incorporates this perspective for art therapy assessment and treatment (Van Meter and Hinz, 2024).

Goals of a neuroscience-based approach to art therapy are threefold. First, an improved understanding of the neural processes that underlie the complex interaction between perception, cognition, emotion, learning, memory, and behavior can lead to an understanding of how therapeutic change occurs, resulting in improved treatment and better outcomes for clients. Second, we can use the data to inform the public about the effectiveness of art therapy, arguably a niche field, to provide accessible, safe, and effective services, and third, this understanding can be used to support the development of hypothesis-based, mechanistic research.

However, it can be tricky to connect art therapy and psychological theory directly to neural responses. Further, high-level cognitive, emotional, and symbolic processing requires complicated interactions involving multiple brain regions (Camacho et al., 2023; Dum et al., 2019; Han et al., 2021; Tseng et al., 2023). There are crucial aspects to consider when applying neuroscience-based frameworks to the practice of art therapy. We need to have a clear understanding of terminology, think critically about data in context, and update our understanding as new data become available, particularly when applying that information to clinical practice. The choice to share information with clients must be made in the context of the individual treatment plan and information must be accurate. People prefer explanations for psychological phenomena that include a neuroscience component, even when the information is irrelevant (Bennett and McLaughlin, 2024; Hopkins et al., 2016; Weisberg et al., 2008, 2018). We need to be wary of oversimplifications, avoid attributing causality to correlational data, and provide accurate, relevant information, even if incorrect or outdated information is attractive. The purposes of this perspective are to provide an overview of the current evidence and limits of neurobiological concepts applied to art therapy practice, and to provide updated information about outdated concepts that are still actively used in clinical practice.

### 1.1 Evidence and limits: neuroplasticity

Brain tissue is composed of neurons and glia. Myelinated axons comprise the white matter and the cell bodies, dendrites, and synapses comprise the gray matter (Kandel et al., 2021). Synaptic plasticity is the activity-dependent change in the size and number of synaptic contacts that result in the brain’s ability to adapt and change in response to experience, called neuroplasticity (Kandel et al., 2021; Pittenger and Duman, 2008). Neuroplasticity can result in measurable anatomical changes such as changes in cortical thickness and/or the amount of gray matter.

Neuroplasticity is thought to be disrupted or impaired in mental health disorders such as anxiety, depression, post-traumatic stress disorder (PTSD), and substance use disorders (Appelbaum et al., 2023; Fuchs et al., 2004) and is associated with reduced density of dendrites and synapses in the cortex (Pittenger and Duman, 2008). That is, the brain’s capacity to reshape its structure and rewire its connections by strengthening or weakening synaptic transmission is impaired.

These data, along with long-term data about the timing and the efficacy of traditional antidepressants, have fundamentally changed our understanding of the causes of depression. The monoamine theory of depression posits that depression is due to depletion of serotonin, norepinephrine, and/or dopamine in the central nervous system. The theory is consistent with the fact that antidepressants elevate the levels of these neurotransmitters. However, the length of time it takes to see antidepressant effects and the lack of evidence of a primary dysfunction of a specific monoamine system in patients with major depressive disorders suggest that traditional antidepressants may work by supporting neuroplasticity rather than reversing monoamine depletion (Artin et al., 2021). The neuroplasticity theory of depression is supported by data that indicate that the treatment response to classic antidepressants, ketamine, and serotonergic psychedelics may be mediated by changes in neuroplasticity resulting from in increased density of dendrites and synaptic connections (Artin et al., 2021; Hess and Gould, 2023; Pittenger and Duman, 2008; Vargas et al., 2023; Wang et al., 2022).

The neuroplasticity theory of depression provides a plausible framework for the effects of art therapy on depression (Dunphy et al., 2018; Jiang et al., 2020; Kaimal et al., 2018). Creativity is associated with changes in functional connectivity and the expression of genes linked to synaptic plasticity (Orwig et al., 2021). Serotoninergic psychedelic use is associated with increased spontaneous creative insights that are accompanied by changes in the connectivity of the default mode network (Mason et al., 2021).

Creativity training and artistic training appear to result in neuroplastic changes in organization, activity, and connectivity in frontal, emotional, and sensory circuits (Schlegel et al., 2015; Sun et al., 2016, 2020), while depression and PTSD are both associated with dysfunctional plasticity and synaptic loss in prefrontal circuits associated with cognition, emotion, and memory (Appelbaum et al., 2023). If neuroplasticity is mechanism that links healing and creativity, then interventions that increase creative engagement might be effective in alleviating depression. The challenge will be in testing whether art therapy results in neuroplastic changes that correlate with reduced symptoms of depression and whether those effects are specific to the art therapy interventions.

### 1.2 Evidence and limits: mirror neuron systems

In the 1990s, researchers at the Università degli Studi di Parma, discovered neurons in the frontal and premotor cortex of macaque monkeys that fired when the monkey performed a movement and when it observed the same movement by another monkey or a human (Kilner and Lemon, 2013). There is evidence that mirror neurons exist in homologous regions of human brains (de la Rosa et al., 2016; Iacoboni and Dapretto, 2006; Kilner and Lemon, 2013), and these systems are involved in movement recognition, selection of movement, and imitation (Iacoboni and Dapretto, 2006; Mukamel et al., 2010). A recent study showed populations of neurons in mouse hypothalamus are active during aggressive behavior and when seeing aggressive behavior of other mice. External activation of those neurons resulted in aggressive behavior, providing direct evidence of mirror neuron systems in social behaviors (Yang et al., 2023).

In humans, mirror systems are thought to transform sensory observations of others’ actions to an internal visceromotor representation of the action goal, such as reaching for a piece of food, to support planning and control of behavior (Rizzolatti and Sinigaglia, 2016). There are correlational and indirect data that that mirror systems underlie the ability to infer the intentions of and emotions of others based on one’s own internal experiences (Heyes and Catmur, 2022; Napolitano, 2021), that motor resonance to stimuli representing social connection positively correlates with empathy scores (Guidali et al., 2023), and that mirror systems support social interaction and learning during childhood development (Dickerson et al., 2017). However, mirror neurons also respond to non-natural movements of inanimate objects (Napolitano, 2021), and the direct evidence that mirror systems are involved in higher level cognitive or empathetic processes is limited (Heyes and Catmur, 2022).

The process of art making is integral to the art therapy process. It is likely that mirror systems are activated when art therapists observe clients’ making art. In fact, it is tempting to speculate that activation of mirror systems may be related to the psychodynamic concept of countertransference. This speculation underscores that caveat that any inferences we make about client behavior, cognition and emotions, even if we frame them as based on mirror systems, still arise from our own internal visceromotor representations, which are based on our own learning, experiences and emotions. Further, positing a mirror neuron system to underlie therapeutic relationship does not change the fact that that mirror systems do not fully explain relational phenomena such as behavioral synchrony and entrainment (Denworth, 2023; Wheatley et al., 2012; Wohltjen et al., 2023), phenomena that may also be important in building therapeutic relationships.

### 1.3 Evidence and limits: interoception and the brain-gut axis

The central nervous system (CNS) receives information about the internal state of the body from the vagus nerve of the parasympathetic nervous system (Breit et al., 2018). The vagus nerve is a mixed nerve, with motor neurons that of the convey parasympathetic motor output to internal organs (~10–20%) and sensory neurons that convey information about the internal state of the body to the brain (~80–90%) (Breit et al., 2018; Paciorek and Skora, 2020). The motor outputs are responsible for the “rest and digest” functions that counterbalance sympathetic “fight or flight” responses. The vagal sensory neurons project to a wide range of brain regions, including brainstem nuclei, the insula, the thalamus, hippocampus, amygdala (Breit et al., 2018; Engelen et al., 2023). Vagal sensory projections give rise to interoception, knowledge about the internal state of the body. Interoception that can occur at several levels. The non-conscious level is associated with homeostatic and allostatic mechanisms and with the sense of self and body ownership. The preconscious level is thought to influence cognition, affective processing, and decision-making. The conscious level includes direct awareness of the body, along with metacognitive awareness of body processes and emotions (Engelen et al., 2023).

Interoception can include sensations such as heartbeat awareness, pain and temperature signals as well as hunger and satiety signals (Breit et al., 2018) that can affect behavior on preconscious or conscious levels, and might presumably be reflected in the art process. Information about and responses to the internal condition of the body can be influenced by gut microbiome. The brain-gut axis can influence regulation of the stress response, immune function, digestion, and heart rate (Palacios-García and Parada, 2020) as well as brain systems involved in mood and anxiety disorders, PTSD, substance use disorders, and eating disorders (Breit et al., 2018; Engelen et al., 2023; Palacios-García and Parada, 2020).

Parasympathetic activity is referred to as vagal tone. Higher vagal tone is thought to be adaptive. It is indirectly measured by heart rate variability (HRV) which is thought to determine relative contributions of the parasympathetic motor system to control of heart rate (Laborde et al., 2017). HRV refers to how much variability occurs in the interval between heartbeats. For example, two individuals may each have a resting heart rate of 60 beats per minute. The individual with one beat exactly every second would have low HRV, while the individual with an interval between beats that varies in a range of a beat every 0.75–1.25 s would have a higher HRV. While there are more than 70 variables that can be considered in measuring HRV, in general, higher HRV is associated with more relative parasympathetic input, greater vagal tone, lower stress levels, and potentially better cognitive and emotional regulation (Laborde et al., 2017).

These data suggest that art therapy interventions that focus on interoceptive processes might be possible therapeutic targets. The activation of central and peripheral systems in art making could plausibly interact with the three levels of interoception, with the potential of using HRV as an outcome measure.

### 1.4 Myth: one region, one function

The first direct evidence of the role of the brain in human behavior was from the loss of specific functions after brain injuries or strokes, demonstrating that a specific region was necessary for a specific function. The early localization of function data led to the first understanding of functional roles of brain regions. For example, the removal of Henry Mollasion’s temporal lobe as a treatment for otherwise intractable epilepsy led to the understanding the different brain regions are necessary for retention of explicit and procedural memories (Baro and Priftis, 2023). Another example is the post-mortem brain damage in patients with aphasia studied by Paul Broca in the 19th century. These studies led to an eponymously named anatomical region, Broca’s area, thought to be necessary for language production (Rutten, 2022). In part because brain function in living humans can now be measured noninvasively by techniques such as electroencephalography (EEG), positron emission tomography (PET), magnetoencephalography (MEG), and functional magnetic resonance imaging (fMRI) (Konopka et al., 2024), these localization of function studies have given way to the understanding that complex cognition and behavior are associated with the activity of multiple cortical and subcortical brain regions organized into complex bilateral networks (Koban et al., 2021). Single brain structures may be necessary for a given function, but they are not sufficient by themselves.

### 1.5 Myth: triune brain

Paul MacLean’s Triune Brain Theory emphasized the hierarchical function of three key brain regions that were said to reflect evolutionary steps from reptiles to early mammals and to late mammals (MacLean, 1982). MacLean’s theory was based in part on Paul Broca’s work showing an evolutionarily conserved anatomical region around the edge of the brain stem, and used the term “limbic” to describe this region (Ploog, 2003). The protoreptilian brain included parts of the midbrain, diencephalon, and basal ganglia and was said to be responsible for instinctive, species-typical, and routine behaviors. The paleomammalian brain represented the transition from retile to mammals and included the limbic system, and was said to be responsible for maternal care, vocal communication, and play. Finally, the neomammalian brain comprised the neocortex and thalamus, and was thought to be the basis of sensory, cognitive, and linguistic processes (Ploog, 2003).

This theory has been discredited because the assumption that each “newer” region was anatomically layered upon the “older” regions reflects an incorrect understanding of evolution (Cesario et al., 2020). In the triune brain formulation, reptilian brains would not have limbic or neocortical regions. However, brain structures are evolutionarily conserved from reptiles to mammals. Instead, the homologous structures differ in size and proportion (Cesario et al., 2020). Steffen’s Adaptive Brain Theory is a more recent alternative evolutionary theory that posits interdependent networks that support adaptation, survival, and reproduction (Steffen et al., 2022). In this conceptualization, the integration of interoceptive and exteroceptive information enables optimal adaptation to continuously changing internal and external environments.

While the triune brain theory has been used to explain and normalize behaviors and emotional responses, the conceptualization of a part of the brain as independently instinctual has the potential to cause harm or impede therapeutic progress. Ascribing behavior related to the autonomic nervous system as controlled by the “reptilian-brain” implies that one has no choice about the “instinctive” behavior. It implies judgments about the behaviors or the person, and in some cases, individuals may use this language to avoid uncomfortable change.

The evolutionary perspective that humans have integrated functions that are adaptive for fast survival responses is important but is better served by an approach that identifies specific structures associated with cognition, behavior, and emotion in context. Using language related to adaptive responses is more consistent with meeting treatment goals around improving coping skills and creating behavioral change.

### 1.6 Myth: right brain vs. left brain

One of the most pernicious myths associated with human creativity is that the “right brain” is exclusively associated with creativity and the “left brain” is exclusively associated with analytical skills. While there are important structural and functional asymmetries (Esteves et al., 2020; Khalil and Demarin, 2023), unless you are a sleeping dolphin (Mascetti, 2021), both hemispheres are active and communicating all the time. The corpus callosum in healthy humans has ~100 million myelinated axons (Goldstein et al., 2023). Severing those connections to treat intractable epilepsy resulted in disconnection syndromes and communication impairments that were overgeneralized in popular culture.

While high creativity is associated with more interhemispheric connectivity (Khalil and Demarin, 2023), data indicate that art-making or creativity can enhance or change connections between neurons in many brain regions (Khalil and Demarin, 2023; Orwig et al., 2021; Schlegel et al., 2015; Sun et al., 2016). Further, creative cognition requires integration of regions and networks across both hemispheres (Patil et al., 2021). The artificial separation of analytic and creative skills, or of science and art, implies that people are only capable of one or the other. This approach can subtly undermine an individual’s sense of capability and self-efficacy, rather than supporting an integrated frame of healing through artmaking and creativity.

## 2 Discussion

The human nervous system is complicated, and researchers are coming to understand that most complex behavior is related to complex patterns of brain activity. Art therapists work with rich, complex behavior, using art created within a therapeutic relationship as a proxy for and communication of internal experience. A neuroscience-based approach provides an avenue for understanding the interplay of sensation, perception, cognition, memory, emotion, and behavior.

There are several neuroscience-based frameworks that provide means of conceptualizing complex behaviors within the art therapy setting and that can be utilized as frameworks for art therapy assessment and intervention (Czamanski-Cohen and Weihs, 2016; Hass-Cohen, 2024; Kaimal, 2019; Lusebrink and Hinz, 2020; Vaisvaser, 2021; Van Meter and Hinz, 2024). Further there are preliminary studies exploring the physiological effects of art making and art therapy using quantitative EEG recordings (Belkofer et al., 2014; Konopka et al., 2024), measurements of salivary cortisol (Kaimal et al., 2016), and brain imaging techniques such as fNIRS (Kaimal et al., 2017; King and Kaimal, 2019) and fMRI (Payano Sosa et al., 2023; Walker et al., 2018) to measure physiological responses to art making and art therapy. Future expansions could also include simple, non-invasive methods such as smartwatches or Fitbits to measure heart rate, HRV, and/or sleep that could be correlated with self-report and art-based qualitative measures to develop testable mechanistic hypotheses and further develop neuroscience-based art therapy frameworks.

## 3 Conclusion

It is important to continually update our understanding by critically appraising and incorporating new data, discarding out of date theories, and implementing new knowledge with care, curiosity, and compassion.

Critical analysis and understanding of the current scientific knowledge base can be used to guide the development of individualized therapeutic goals within art therapy practice and support the development of hypothesis-based research to determine the primary mechanisms that drive the observed effects of art therapy interventions.

## Data Availability

The original contributions presented in the study are included in the article/supplementary material, further inquiries can be directed to the corresponding author.
